# Cardiac Tamponade as the Initial Presentation of Acute Myeloid Leukemia: A Case Report with Review of the Literature

**DOI:** 10.1155/2018/8201917

**Published:** 2018-03-26

**Authors:** Dillon Karmo, Adam Hafeez, Alexandra Halalau, Siddhartha Yadav

**Affiliations:** ^1^Department of Internal Medicine, Beaumont Hospital, Royal Oak, MI 48073, USA; ^2^Oakland University William Beaumont School of Medicine, Rochester, MI 48073, USA; ^3^Department of Hematology-Oncology, Mayo Clinic School of Graduate Medical Education, Mayo Clinic, 200 1st St. SW, Rochester, MN 55905, USA

## Abstract

Acute myeloid leukemia (AML) is a complex disease with a variety of presentations. A large pericardial effusion is rare, occurring in less than 0.5% of all patients with AML prior to treatment. A 34-year-old male presented with dyspnea, malaise, and weight loss. On physical exam, he was noted to be hypoxic, tachypneic, tachycardic, and hypotensive. He had cervical lymphadenopathy and jugular venous distention. His WBC count was 110 bil/L with 33% blasts. Bone marrow biopsy confirmed AML with 60% blasts. Leukemic cells were also seen in the cerebrospinal fluid on lumbar puncture. An echocardiogram revealed a large pericardial effusion causing tamponade. He underwent emergent pericardiocentesis, and malignant cells were present in the pericardial fluid. Induction therapy with standard dose cytarabine and daunorubicin was initiated, and bone marrow biopsy 14 days later showed no residual AML. This case demonstrates the importance of a thorough evaluation of each organ system when caring for a patient with AML.

## 1. Introduction

Acute myeloid leukemia (AML) is a complex disease with a variety of presentations. Majority of patients present with symptoms related to bone marrow failure such as fatigue, bruising or bleeding, fever, and infection [1]. Pericardial effusion can occur in patients with AML, but a large pericardial effusion is rare, occurring in less than 0.5% of all patients with AML prior to treatment [2]. Cardiac tamponade in AML is exceedingly rare, with only few cases reported in the literature. In this report, we describe a case of AML with cardiac tamponade as the initial presentation.

## 2. Case Description

**Figure 1:**
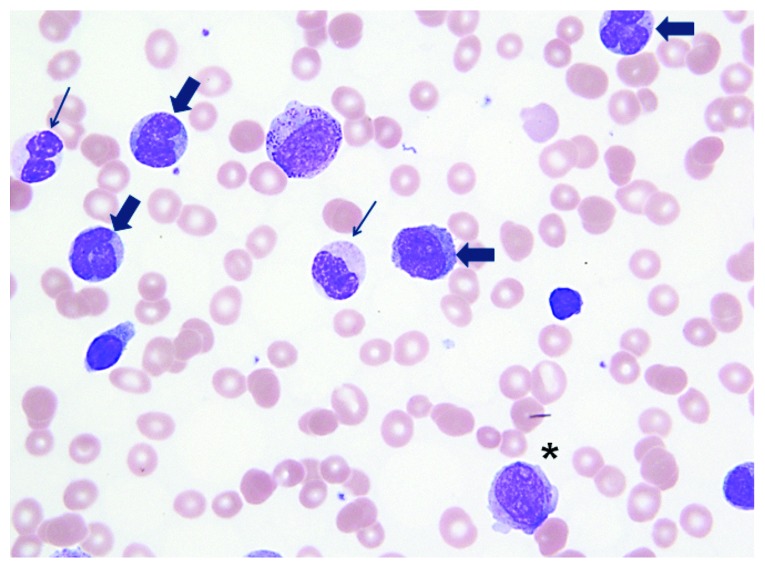
Peripheral smear: Giemsa stain (1000x). Thin arrows denote hypogranular granulocytes, thick arrows denote monocytes, and ^∗^denotes circulating myeloblasts.

A 34-year-old male without any significant past medical history presented to the hospital with shortness of breath, malaise, and unintentional weight loss. On physical exam, he was noted to be hypoxic, tachypneic, tachycardic, and hypotensive. He had palpable cervical lymphadenopathy and jugular venous distention. Complete blood count showed hyperleukocytosis (white blood cell count (WBC) of 110 bil/L with 33% blasts) and severe anemia (hemoglobin of 4.0 g/dL). Peripheral blood smear demonstrated increased blasts with promonocytes consistent with AML (Figure 1). His EKG showed sinus tachycardia and low-voltage QRS.

**Figure 2:**
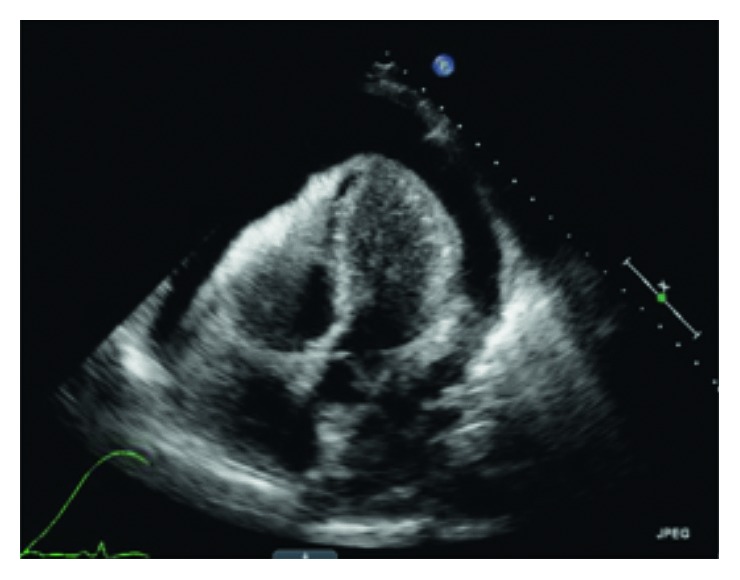
Echocardiogram showing large pericardial effusion with right atrial and ventricular collapse.

**Figure 3:**
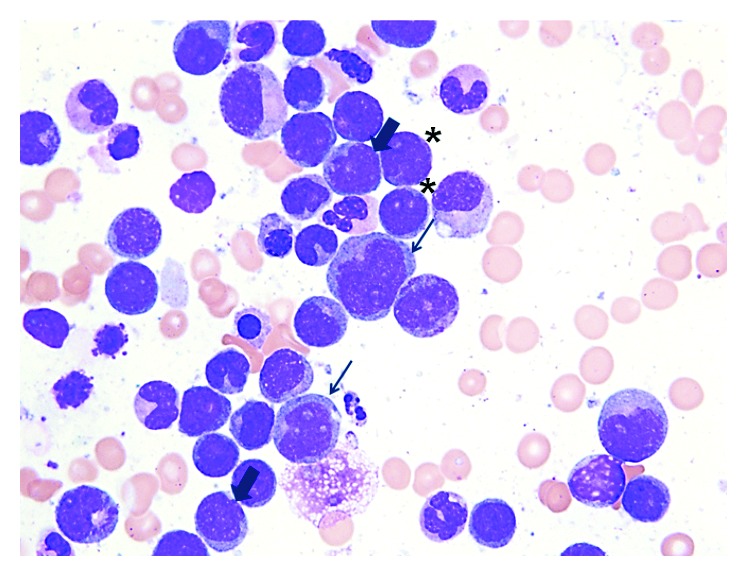
Bone marrow aspirate, 1000x, Giemsa stain. ^∗^denotes myeloblasts, thin arrows denote the monoblasts, and thick arrows denote promonocytes.

Given his dyspnea, hemodynamic abnormalities, and jugular venous distension, an emergent echocardiogram was obtained which revealed a large pericardial effusion with right atrial and ventricular collapse consistent with cardiac tamponade (Figure 2). Pericardiocentesis was performed, and leukemic blasts were seen on fluid analysis. Despite a lack of neurological symptoms, a lumbar puncture was performed as he was deemed high risk for central nervous system (CNS) involvement due concern for acute myelomonocytic leukemia (AMML). Cerebrospinal fluid analysis revealed 99 WBCs with 35% blasts consistent with leukemic involvement. Bone marrow biopsy was consistent with AMML (Figure 3). Cytogenetics revealed no abnormalities. On genotyping, NPM1, FLT-3, CEBP-A, and C-KIT were negative.

His dyspnea, tachypnea, tachycardia, hypotension, and hypoxia resolved following pericardiocentesis. Given his hemodynamic instability, treatment with standard dose 7 + 3 cytarabine with daunorubicin was preferred over high-dose cytarabine with daunorubicin.

The pericardial effusion did not reaccumulate, and he did not require a repeat pericardiocentesis. Bone marrow biopsy performed on day 14 of treatment did not show any residual AML. His blood counts recovered to normal, and he was started on intrathecal methotrexate. He is currently undergoing bone marrow transplant evaluation.

## 3. Discussion

Pericardial tamponade as an initial presentation of AML is a rare entity. A thorough literature review identified only three prior reported such cases in adults [3–5]. The clinical presentations of these cases have been summarized in Table 1. Two of the three patients described in the literature (Table 1) died within 24 hours. In a prior study evaluating 1600 patients with different types of leukemia, it is described that pericardial effusion was detected in 21% of patients with AML [2]. In more than two-thirds of these patients, the effusions occurred after receiving some form of therapy, whereas the remainders of effusions were present before therapy, and none was the initial presentation.

Approximately half of patients with symptomatic pericardial disease and an underlying cancer may have nonmalignant pericardial disease including radiation pericarditis, drug-induced pericarditis, infection, hypothyroidism, autoimmune disorders, and idiopathic pericarditis [6]. In our case, we were able to establish AML as the clear culprit behind the pericardial effusion by demonstrating blast cells in the pericardial fluid. It is important to consider these other causes of pericardial effusion when such a clear relationship between the malignancy and the pericardial effusion cannot be established.

Although poorly understood, the pathophysiology behind the relationship between leukemia and acute pericardial disease is thought to be related to multiple factors including hemorrhage caused by concurrent thrombocytopenia, infections due to underlying immune deficiency, and direct malignant cell infiltration [7, 8]. While the prognostic and therapeutic implications of malignant effusions in AML are not entirely known, it appears that an effusion large enough to cause tamponade increases mortality.

Our case also highlights the importance of evaluating for CNS involvement in patients with AML. Central nervous system (CNS) involvement occurs in around 0.6% of adult patients with AML at initial presentation [9]. These patients may not have any CNS symptoms [10], as was seen in our case as well. Not all patients with AML require a lumbar puncture, but the decision to perform a lumbar puncture should not only rest on CNS symptoms but also on the presence of other risk factors for CNS involvement such as prominent monocytic component, acute promyelocytic leukemia, and certain molecular and cytogenetic findings among many others [9–12].

## 4. Conclusions

In conclusion, our case demonstrates the importance of a thorough evaluation of each organ system when caring for a patient with AML. Pericardial effusion causing cardiac tamponade can be one of the initial presentations of AML and requires early recognition and prompt intervention to improve outcome.

## Figures and Tables

**Table 1:** Characteristics of previously reported cases of AML with cardiac tamponade on presentation.

Patient demographics	Signs of tamponade	Chemotherapeutic regimen	Outcome
32-year-old male [3]	Present	None	Patient died within 24 hours of admission
28-year-old male [4]	Present	Intrapericardial mitoxantrone	Complete remission
73-year-old female [5]	None	None	Patient died prior to pericardiocentesis
